# mTOR Inhibitors Can Enhance the Anti-Tumor Effects of DNA Vaccines through Modulating Dendritic Cell Function in the Tumor Microenvironment

**DOI:** 10.3390/cancers11050617

**Published:** 2019-05-02

**Authors:** Yu-Li Chen, Han-Wei Lin, Nai-Yun Sun, Jr-Chi Yie, Hsueh-Chih Hung, Chi-An Chen, Wei-Zen Sun, Wen-Fang Cheng

**Affiliations:** 1 Department of Obstetrics and Gynecology, College of Medicine, National Taiwan University, Taipei City 100, Taiwan; uly1007@yahoo.com.tw (Y.-L.C.); r05423018@ntu.edu.tw (H.-C.H.); chianchen@ntu.edu.tw (C.-A.C.); wenfangcheng@yahoo.com (W.-F.C.); 2 Graduate Institute of Oncology, College of Medicine, National Taiwan University, Taipei City 100, Taiwan; handway_rc@hotmail.com (H.-W.L.); naiyunsun@gmail.com (N.-Y.S.); 3 Department of Medicine, National Taiwan University Hospital Jin-Shan Branch, New Taipei City 20844, Taiwan; yiejrchi@gmail.com; 4 Department of Anesthesiology, College of Medicine, National Taiwan University, Taipei City 100, Taiwan; 5 Graduate Institute of Clinical Medicine, College of Medicine, National Taiwan University, Taipei City 100, Taiwan

**Keywords:** mTOR inhibitor, DNA vaccine, dendritic cells, tumor microenvironment

## Abstract

The life span of dendritic cells (DCs) can become short following induced activation, which is associated with metabolic transition due to the regulation of mechanistic target of rapamycin (mTOR). The purpose of this study was to investigate the potential of inhibiting mTOR to modulate DC functions for elevating the anti-tumor effects of DNA vaccines. Therefore, the influences of various inhibitors of mTOR (mTORi) on the expressions of DC maturation markers, the abilities of antigen presenting and processing of BMM-derived DCs and the tumor killing effects of E7-specific CD8^+^ T lymphocytes activated by BMM-derived DCs were in vitro examined. The anti-tumor effects of connective tissue growth factor (CTGF)/E7 DNA vaccine and/or mTORi were also in vivo analyzed. In our study, suppressive effects of mTORi on the DC maturation markers expressed on BMMCs could be reversed. The mTORi-treated mature BMM-derived DCs tended to be non-apoptotic. These mTORi-treated BMM-derived DCs could have better antigen presenting and processing abilities. The E7-specific cytotoxic CD8+ T lymphocytes could have more potent tumoricidal activity following activation of mTORi-treated BMM-derived DCs. For tumor-bearing mice, those treated with CTGF/E7 DNA vaccine and mTORi indeed can have higher percentages of mature DCs in the TME, better disease control and longer survivals. Consequently, application of mTORi can be a pharmacological approach for temporally increasing life span, antigen presenting and antigen processing of DCs to strengthen the therapeutic outcome of cancer immunotherapy.

## 1. Introduction

Cancer treatment has been revolutionized the past two decades by the advanced development of immunotherapy in. One of the key coordinators responsible for host anti-tumor immunities is dendritic cells (DCs). DCs are the most potent antigen-presenting cells for processing tumor antigens and priming different subsets of antigen-specific T cells. These cells also can play a critical role at the interface between the innate and adaptive arms of the immune system [1,2]. However, the decreased effectiveness of DC-induced anti-tumor responses in tumor-bearing hosts can be resulted from multiple factors. These reasons include low number of DCs in the tumor microenvironment (TME), poor access of DCs to tumor antigens, limited capacity of tumor cells to stimulate intratumoral DCs [3,4,5], short life span following induced DC maturation [6,7], and secretion of cytokines by the tumor cells that suppress DC maturation [8,9].

When DCs are induced to be mature by toll-like receptor (TLR) agonists, the life span of DCs would become short [6,7]. The possible reason can be that metabolism of mouse DCs shifts from oxidative phosphorylation towards aerobic glycolysis. These DCs are glucose-dependent and able to rapidly exhaust available glucose or other nutrients in tissue culture medium following stimulation by TLR agonists [10]. Previous studies have documented that high levels of glucose consumption generally correlate with shorter life span and this regulation of glycolytic metabolism is associated with mechanistic target of rapamycin (mTOR) [11,12]. Restriction of glucose usage and caloric intake can lead to increased cellular and organismal longevity [13,14]. In addition, several studies have demonstrated that inhibition of mTOR can extend the life span of eukaryotic cells and organisms [15,16,17].

Therefore, we hypothesized that mTOR can play a regulatory role in controlling DC functions for enhancing the anti-tumor immunities of tumor-bearing hosts based on these evidences. The impacts of various inhibitors of mTOR (mTORi) on the DC maturation markers expressed on bone marrow mononuclear cells (BMMCs), immature bone marrow monocyte (BMM)-derived DCs or mature BMM-derived DCs in the process of developing DCs from BMMCs were first in vitro examined. Then, the abilities of antigen presenting and processing of BMM-derived DCs and the tumoricidal activities of E7-specific CD8^+^ T lymphocytes activated by BMM-derived DCs were further evaluated in the presence of mTORi. Finally, we in vivo analyzed the anti-tumor effects of connective tissue growth factor (CTGF)/E7 DNA vaccine and/or mTORi. In addition to the host immune responses, the distributions of mature DCs in the draining lymph nodes and TME were investigated in tumor-bearing mice treated with various treatment modalities.

## 2. Results

### 2.1. Suppressive Effects of mTORi on the DC Maturation Markers Expressed on BMMCs Could Be Reversed

**Figure 1:**
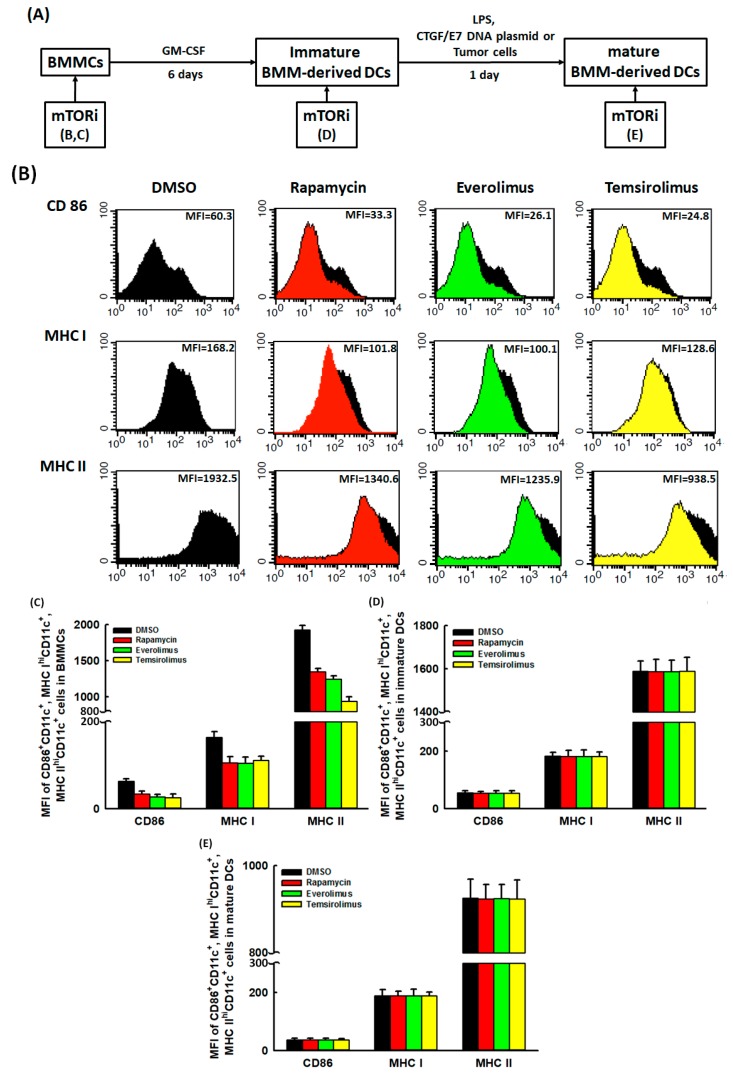
Influence of mTORi on the DC maturation markers expressed on BMMCs, immature BMM-derived DCs and mature BMM-derived DCs. (**A**) Schematic diagram showing the process of in vitro developing DCs from murine BMMCs. (**B**) Representative figures of flow cytometric analyses for the MFIs of CD86^+^CD11c^+^, MHC I^hi^CD11c^+^, and MHC II^hi^CD11c^+^ cells in BMMCs treated with/without mTORi. (*n* = 3) (**C**) Bar figures showed the MFIs of CD86^+^CD11c^+^, MHC I^hi^CD11c^+^, and MHC II^hi^CD11c^+^ cells in BMMCs treated with/without mTORi. The MFIs of CD86^+^CD11c^+^ (*p* = 0.016), MHC I^hi^CD11c^+^ (*p* = 0.024), and MHC II^hi^CD11c^+^ (*p* = 0.018) cells were lower in mTORi-treated BMMCs than those in non-mTORi-treated groups. (*n* = 3) (**D**) Bar figures showed the MFIs of CD86^+^CD11c^+^, MHC I^hi^CD11c^+^, and MHC II^hi^CD11c^+^ cells in immature BMM-derived DCs treated with/without mTORi. The MFIs of CD86^+^CD11c^+^, MHC I^hi^CD11c^+^, and MHC II^hi^CD11c^+^ cells did not show differences between mTORi-treated immature BMM-derived DCs and non-mTORi-treated groups. (*n* = 3) (**E**) Bar figures showed the MFIs of CD86^+^CD11c^+^, MHC I^hi^CD11c^+^, and MHC II^hi^CD11c^+^ cells in mature BMM-derived DCs treated with/without mTORi. The MFIs of CD86^+^CD11c^+^, MHC I^hi^CD11c^+^, and MHC II^hi^CD11c^+^ cells did not show differences between mTORi- and non-mTORi-treated mature BMM-derived DCs. (*n* = 3)

The process of in vitro developing DCs from murine BMMCs was shown in Figure 1A. To investigate the influence of mTORi on the alterations of DC maturation markers in this process, the mean fluorescent intensities (MFIs) of CD86^+^CD11c^+^, major histocompatibility complex (MHC) I^hi^CD11c^+^, and MHC II^hi^CD11c^+^ cells in BMMCs, immature BMM-derived DCs or mature BMM-derived DCs treated with/without mTORi were first analyzed by flow cytometry. Figure 1B showed the representative MFIs of CD86^+^CD11c^+^, MHC I^hi^CD11c^+^, and MHC II^hi^CD11c^+^ cells in BMMCs treated with/without mTORi. The MFIs of CD86^+^CD11c^+^ (*p* = 0.016), MHC I^hi^CD11c^+^ (*p* = 0.024), and MHC II^hi^CD11c^+^ (*p* = 0.018) cells were lower in mTORi-treated BMMCs than those in non-mTORi-treated groups (Figure 1C). However, the MFIs of CD86^+^CD11c^+^, MHC I^hi^CD11c^+^, and MHC II^hi^CD11c^+^ cells did not show differences between mTORi-treated immature BMM-derived DCs and non-mTORi-treated groups (Figure 1D). Similar phenomena were observed between mTORi-treated mature BMM-derived DCs and non-mTORi-treated groups (Figure 1E). Therefore, mTORi could have suppressive impacts on the DC maturation markers expressed on BMMCs.

**Figure 2:**
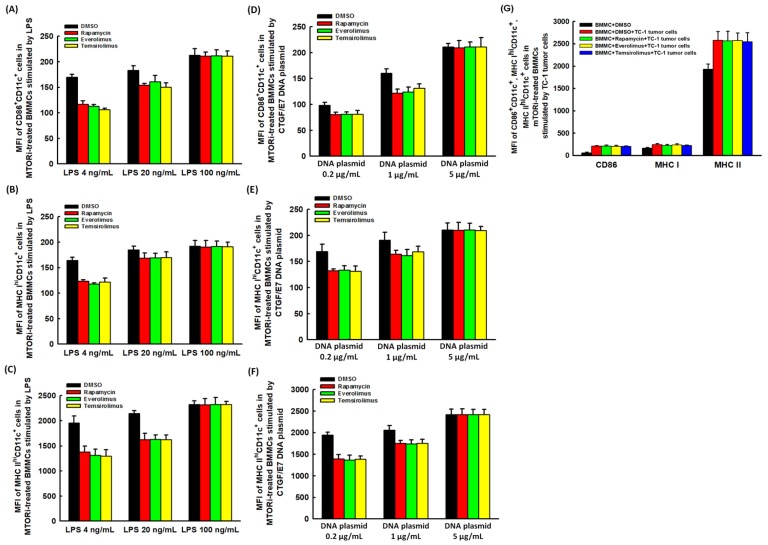
DC maturation markers expressed on mTORi-treated BMMCs could be modulated. (**A**–**C**) Bar figures exhibited the MFIs of CD86^+^CD11c^+^ (**A**), MHC I^hi^CD11c^+^ (**B**), and MHC II^hi^CD11c^+^ (**C**) cells in mTORi-treated BMMCs stimulated by LPS. Lower MFIs of CD86^+^CD11c^+^, MHC I^hi^CD11c^+^, and MHC II^hi^CD11c^+^ cells in mTORi-treated BMMCs could be elevated by LPS with positive dose response relationship. (*n* = 3) (**D**–**F**) Bar figures exhibited the MFIs of CD86^+^CD11c^+^ (**D**), MHC I^hi^CD11c^+^ (**E**), and MHC II^hi^CD11c^+^ (**F**) cells in mTORi-treated BMMCs stimulated by CTGF/E7 DNA plasmid. Lower MFIs of CD86^+^CD11c^+^, MHC I^hi^CD11c^+^, and MHC II^hi^CD11c^+^ cells in mTORi-treated BMMCs could be elevated by CTGF/E7 DNA plasmid with positive dose response. (*n* = 3) (**G**) Bar figures exhibited the MFIs of CD86^+^CD11c^+^, MHC I^hi^CD11c^+^, and MHC II^hi^CD11c^+^ cells in mTORi-treated BMMCs stimulated by TC-1 tumor cells. Lower MFIs of CD86^+^CD11c^+^, MHC I^hi^CD11c^+^, and MHC II^hi^CD11c^+^ cells in mTORi-treated BMMCs could be increased by TC-1 tumor cells at the ratio of BMMC:TC-1 = 1:1. (*n* = 3).

However, the lower MFIs of CD86^+^CD11c^+^, MHC I^hi^CD11c^+^, and MHC II^hi^CD11c^+^ cells in mTORi-treated BMMCs could be elevated by lipopolysaccharides (LPS) (Figure 2A–C) and CTGF/E7 DNA plasmid (Figure 2D–F) with positive dose response relationship. In addition, the lower MFIs of CD86^+^CD11c^+^, MHC I^hi^CD11c^+^, and MHC II^hi^CD11c^+^ cells in mTORi-treated BMMCs could be increased by TC-1 cells at the ratio of BMMC:TC-1 = 1:1 (Figure 2G). Therefore, the suppressive effects of mTORi on the DC maturation markers expressed on BMMCs could be reversed by the stimulation of LPS, CTGF/E7 DNA plasmid and TC-1 tumor cells.

### 2.2. Mature BMM-Derived DCs Treated with mTORi Tended to Be Non-Apoptotic

**Figure 3:**
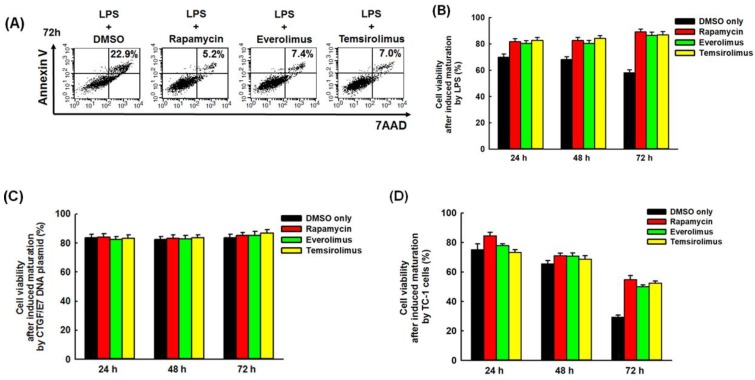
Anti-apoptotic effects of mTORi on mature BMM-derived DCs. (**A**) Representative figures of flow cytometric analyses for the percentages of apoptotic cells (Annexin V^+^7AAD^+^ cells) in mature BMM-derived DCs in vitro induced by LPS and then treated with/without mTORi. The percentages of apoptotic cells were lower in mTORi-treated groups than those in non-mTORi-treated group. (*n* = 3) (**B**) Bar figures showed the percentages of non-apoptotic cells (Annexin V^−^7AAD^−^ cells) in mature BMM-derived DCs in vitro induced by LPS and then treated with/without mTORi. The percentages of non-apoptotic cells decreased with time in non-mTORi-treated group (*p* = 0.027, *n* = 3) and were lower in non-mTORi-treated than those in mTORi-treated groups at indicated intervals (72 h: *p* = 0.019, *n* = 3). The percentages of non-apoptotic cells did not decrease with time and were all more than 80% in mTORi-treated groups. (**C**) Bar figures showed the percentages of non-apoptotic cells in mature BMM-derived DCs in vitro induced by CTGF/E7 DNA plasmid and then treated with/without mTORi. The percentages of non-apoptotic cells did not alter with time and were similar at indicated intervals in non-mTORi-treated and mTORi-treated groups. The percentages of non-apoptotic cells were all more than 80% in both groups. (*n* = 3) (**D**) Bar figures showed the percentages of non-apoptotic cells in mature BMM-derived DCs in vitro induced by TC-1 tumor cells and then treated with/without mTORi. The percentages of non-apoptotic cells decreased with time in non-mTORi-treated group (*p* = 0.025, *n* = 3) and mTORi-treated groups (Rapamycin: *p* = 0.023, *n* = 3). The percentages of non-apoptotic cells were higher in mTORi-treated group than those in non-mTORi-treated groups at indicated intervals (72 h: *p* = 0.038, *n* = 3).

Figure 3A representatively demonstrated flow cytometric percentages of apoptotic cells [Annexin V^+^7-amino-actinomycin D (7AAD)^+^ cells] in mature BMM-derived DCs in vitro induced by LPS and then treated with/without mTORi. The percentages of apoptotic cells were lower in mTORi-treated groups than those in non-mTORi-treated group. The percentages of non-apoptotic cells (Annexin V^−^7AAD^−^ cells) in mature BMM-derived DCs in vitro induced by LPS decreased with time in non-mTORi-treated group (24 h: 69.9 ± 2.4%; 48 h: 68.1 ± 2.1%; 72 h: 58.1 ± 2.3%; *p* = 0.027,) and were lower in non-mTORi-treated group than those in mTORi-treated groups at indicated intervals (72 h: DMSO, 58.1 ± 2.3%; rapamycin, 89.1 ± 2.2%; everolimus, 86.5 ± 2.4%; temsirolimus, 86.9 ± 2.4%; *p* = 0.019, Figure 3B). The percentages of non-apoptotic cells did not decrease with time and were all more than 80% in mTORi-treated groups (Figure 3B).

The percentages of non-apoptotic cells in mature BMM-derived DCs in vitro induced by CTGF/E7 DNA plasmid did not alter with time and were similar at indicated intervals in non-mTORi-treated and mTORi-treated groups (Figure 3C). The percentages of non-apoptotic cells were all more than 80% in both groups (Figure 3C). The percentages of non-apoptotic cells in mature BMM-derived DCs in vitro induced by TC-1 tumor cells decreased with time in non-mTORi-treated group (24 h: 75.2 ± 3.8%; 48 h: 65.5 ± 2.2%; 72 h: 29.4 ± 1.4%; *p* = 0.025,) and mTORi-treated groups (Rapamycin: 24 h, 84.5 ± 2.4%; 48 h, 71.1 ± 1.6%; 72 h, 54.8 ± 2.8%; *p* = 0.023, Figure 3D). However, the percentages of non-apoptotic cells were still higher in mTORi-treated group than those in non-mTORi-treated groups at indicated intervals (72 h: DMSO, 29.4 ± 1.4%; Rapamycin, 54.8 ± 2.8%; Everolimus, 50.1 ± 1.2%; Temsirolimus, 52.4 ± 1.5%; *p* = 0.038, Figure 3D). These results showed that mature BMM-derived DCs induced by LPS or TC-1 tumor cells and then treated with mTORi tended to be non-apoptotic.

### 2.3. mTORi-Treated BMM-Derived DCs Could Have Better Abilities of Antigen Presenting and Processing

**Figure 4:**
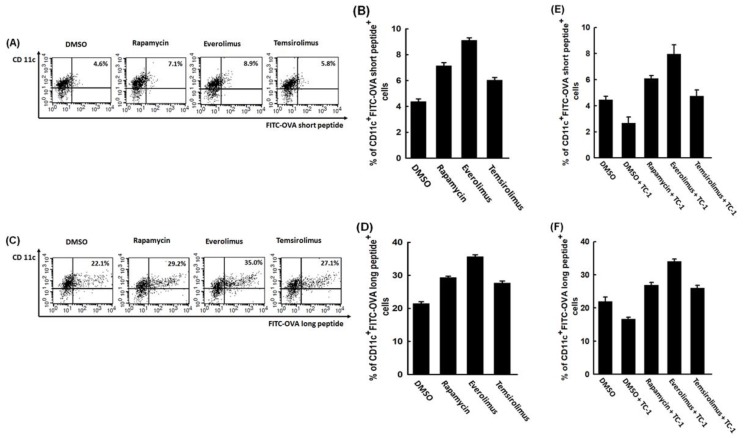
Abilities of antigen presenting and processing of mTORi-treated BMM-derived DCs pulsed with MHC-compatible respective OVA peptide. (**A**) Representative figures of antigen presenting activity of non-mTORi- and mTORi-treated BMM-derived DCs pulsed with FITC-conjugated OVA_257-264_ short peptide by flow cytometric analysis. (*n* = 3) (**B**) Bar figure of the antigen presenting activity of non-mTORi- and mTORi-treated BMM-derived DCs pulsed with FITC-conjugated OVA short peptide. The abilities of antigen presenting were higher in mTORi-treated BMM-derived DCs than non-mTORi-treated group (*p* = 0.017, *n* = 3) (**C**) Representative figures of antigen processing activity of non-mTORi- and mTORi-treated BMM-derived DCs pulsed with FITC-conjugated OVA_323-339_ long peptide by flow cytometric analysis. (*n* = 3) (**D**) Bar figure of the antigen processing activity of non-mTORi- and mTORi-treated BMM-derived DCs pulsed with FITC-conjugated OVA long peptide. The abilities of antigen processing were higher in mTORi-treated BMM-derived DCs than non-mTORi-treated group (*p* = 0.013, *n* = 3) (**E**) Bar figure of the antigen presenting activity of non-mTORi- and mTORi-treated BMM-derived DCs co-cultured with TC-1 tumor cells and then pulsed with FITC-conjugated OVA short peptide. The abilities of antigen presenting of non-mTORi-treated BMM-derived DCs co-cultured with TC-1 tumor cells and then pulsed with FITC-conjugated OVA short peptide would be lower than those only pulsed with FITC-conjugated OVA short peptide (*p* = 0.043, *n* = 3). The abilities of antigen presenting of mTORi-treated BMM-derived DCs co-cultured with TC-1 tumor cells and then pulsed with FITC-conjugated OVA short peptide were still better than those of non-mTORi-treated BMM-derived DCs only pulsed with FITC-conjugated OVA short peptide (*p* = 0.023, *n* = 3). (**F**) Bar figure of the antigen processing activity of non-mTORi- and mTORi-treated BMM-derived DCs co-cultured with TC-1 tumor cells and then pulsed with FITC-conjugated OVA long peptide. The abilities of antigen processing of non-mTORi-treated BMM-derived DCs co-cultured with TC-1 tumor cells and then pulsed with FITC-conjugated OVA long peptide would be lower than those only pulsed with FITC-conjugated OVA long peptide (*p* = 0.048, *n* = 3). The abilities of antigen processing of mTORi-treated BMM-derived DCs co-cultured with TC-1 tumor cells and then pulsed with FITC-conjugated OVA long peptide were still better than those of non-mTORi-treated BMM-derived DCs only pulsed with FITC-conjugated OVA long peptide (*p* = 0.012, *n* = 3).

Figure 4A exhibited representative antigen presenting activity of non-mTORi- and mTORi-treated BMM-derived DCs pulsed with FITC-conjugated OVA_257-264_ short peptide by flow cytometric analysis. The abilities of antigen presenting were higher in mTORi-treated BMM-derived DCs than non-mTORi-treated group (DMSO, 4.4 ± 0.2%; rapamycin, 7.1 ± 0.3%; everolimus, 9.1 ± 0.2%; temsirolimus, 6.0 ± 0.2%; *p* = 0.017, Figure 4B). 

Figure 4C showed representative antigen processing activity of non-mTORi- and mTORi-treated BMM-derived DCs pulsed with FITC-conjugated OVA_323–339_ long peptide by flow cytometry. The abilities of antigen processing were higher in mTORi-treated BMM-derived DCs than non-mTORi-treated group (DMSO, 21.4 ± 0.6%; rapamycin, 29.3 ± 0.5%; everolimus, 36.6 ± 0.6%; temsirolimus, 27.2 ± 0.6%; *p* = 0.013, Figure 4D).

The antigen presenting abilities of non-mTORi-treated BMM-derived DCs co-cultured with TC-1 tumor cells and then pulsed with FITC-conjugated OVA short peptide would be lower than those only pulsed with FITC-conjugated OVA short peptide (DMSO, 4.5 ± 0.3%; DMSO+TC-1, 2.7 ± 0.5%; *p* = 0.043, Figure 4E). The abilities of antigen presenting of mTORi-treated BMM-derived DCs co-cultured with TC-1 tumor cells and then pulsed with FITC-conjugated OVA short peptide were still better than those of non-mTORi-treated BMM-derived DCs only pulsed with FITC-conjugated OVA short peptide (DMSO, 4.5 ± 0.3%; rapamycin+TC-1, 6.1 ± 0.2%; everolimus+TC-1, 8.0 ± 0.7%; temsirolimus+TC-1, 4.7 ± 0.5%; *p* = 0.023, Figure 4E).

The antigen processing abilities of non-mTORi-treated BMM-derived DCs co-cultured with TC-1 tumor cells and then pulsed with FITC-conjugated OVA long peptide would be lower than those only pulsed with FITC-conjugated OVA long peptide (DMSO, 22.0 ± 1.3%; DMSO+TC-1, 16.7 ± 0.6%; *p* = 0.048, Figure 4F). The abilities of antigen processing of mTORi-treated BMM-derived DCs co-cultured with TC-1 tumor cells and then pulsed with FITC-conjugated OVA long peptide were still better than those of non-mTORi-treated BMM-derived DCs only pulsed with FITC-conjugated OVA long peptide (DMSO, 22.0 ± 1.3%; rapamycin+TC-1, 26.9 ± 0.8%; everolimus+TC-1, 34.1 ± 0.7%; temsirolimus+TC-1, 26.1 ± 0.8%; *p* = 0.012, Figure 4F). Consequently, mTORi-treated BMM-derived DCs could have better antigen presenting and processing activities.

### 2.4. In Vitro Tumoricidal Activity of E7-Specific Cytotoxic CD8^+^ T Lymphocytes Could Be Increased by mTORi-Treated BMM-Derived DCs

**Figure 5:**
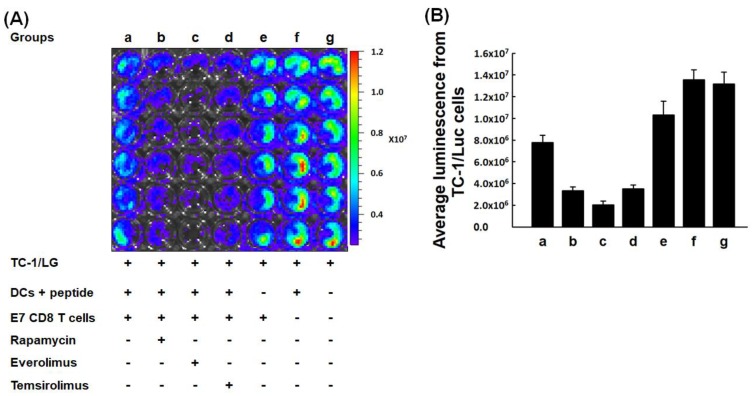
In vitro tumoricidal activity of E7-specific cytotoxic CD8^+^ T lymphocytes activated by mTORi-treated BMM-derived DCs. (**A**) Representative figures of the tumor killing abilities of E7-specific CD8^+^ T lymphocytes activated by non-mTORi- and mTORi-treated BMM-derived DCs using the IVIS system. (*n* = 3) (**B**) Quantification of luminescence of in vitro tumor killing abilities of E7-specific CD8^+^ T lymphocytes activated by non-mTORi- and mTORi-treated BMM-derived DCs. Compared with the luminescence of TC-1/Luc cells co-cultured with E7-specific CD8^+^ T lymphocytes activated by non-mTORi-treated BMM-derived DCs, less luminal activity was detected in TC-1/Luc cells co-cultured with E7-specific CD8^+^ T lymphocytes activated by mTORi-treated BMM-derived DCs (*p* < 0.001, *n* = 3).

Figure 5 demonstrated representative tumor killing activities of E7-specific CD8^+^ T lymphocytes activated by non-mTORi- and mTORi-treated BMM-derived DCs using the IVIS system.

Compared with the luminescence of TC-1/Luc cells co-cultured with E7-specific CD8^+^ T lymphocytes activated by non-mTORi-treated BMM-derived DCs, less luminal activity was detected in TC-1/Luc cells co-cultured with E7-specific CD8^+^ T lymphocytes activated by mTORi-treated BMM-derived DCs (Group a, 7.8 ± 0.7 × 10^6^; Group b, 3.3 ± 0.4 × 10^6^; Group c, 2.0 ± 0.3 × 10^6^; Group d, 3.5 ± 0.4 × 10^6^; Group e, 1.0 ± 0.1 × 10^7^; Group f, 1.4 ± 0.09 × 10^7^; Group g, 1.3 ± 0.1 × 10^7^; *p* < 0.001, Figure 5B). Therefore, in vitro tumoricidal abilities of E7-specific cytotoxic CD8^+^ T lymphocytes could be strengthened by mTORi-treated BMM-derived DCs.

### 2.5. Expression of Apoptotic Molecules, Bad or Bak Can Be Inhibited in BMM-Derived DCs Treated with mTORi

**Figure 6:**
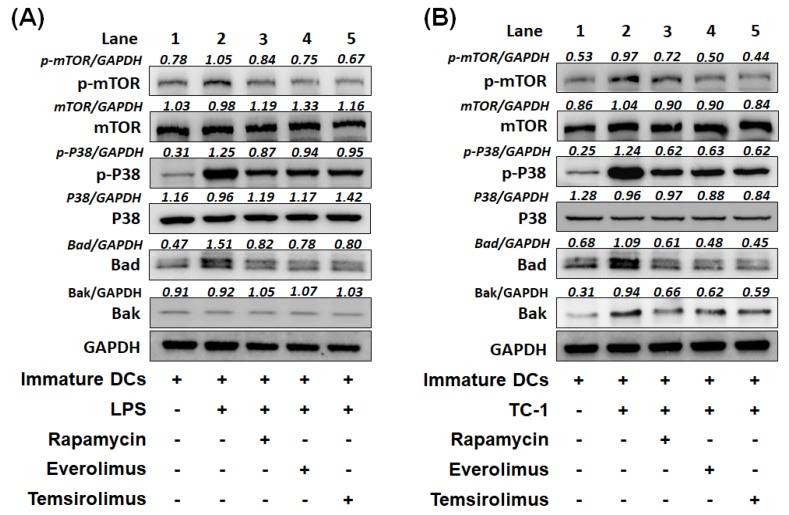
Western blotting of signaling pathways of BMM-derived DCs treated with mTORi. (**A**) The representative signaling pathways in immature BMM-derived DCs treated with or without mTORi and then stimulated by LPS. Compared with non-mTORi-treated BMM-derived DCs, BMM-derived DCs treated with mTORi showed the inhibited phosphorylation of mTOR and p38 and decreased expression of Bad. (*n* = 3) (**B**) The representative signaling pathways in immature BMM-derived DCs treated with or without mTORi and then co-cultured with TC-1 tumor cells. Compared with non-mTORi-treated BMM-derived DCs, mTORi-treated BMM-derived DCs showed the inhibition of phosphorylation of mTOR and p38 and decreased expression of Bad and Bak. (*n* = 3).

We further elucidated the possible signaling molecules involved in the immature BMM-derived DCs treated with/without mTORi and then stimulated by LPS or TC-1 tumor cells. Under the stimulation of LPS, the increased phosphorylation of mTOR and p38 and up-regulated expression of apoptotic molecule, Bad were noted in non-mTORi-treated BMM-derived DCs (Figure 6A, lane 2). In the mTORi-treated groups, the phosphorylation of mTOR and p38 were inhibited and the expression of Bad was decreased (Figure 6A, lanes 3–5).

Under the stimulation of TC-1 tumor cells, the phosphorylation of mTOR and p38 were activated and expression of apoptotic molecules, Bad and Bak were up-regulated in non-mTORi-treated BMM-derived DCs (Figure 6B, lane 2). When BMM-derived DCs treated with mTORi, the phosphorylation of mTOR and p38 were inhibited. The upregulation of Bad and Bak were demolished in the mTORi-treated BMM-derived DCs (Figure 6B, lanes 3–5). Consequently, mTORi can decrease the expression of apoptotic molecules, Bad or Bak in BMM-derived DCs and correlate with the inhibited phosphorylation of mTOR and p38.

### 2.6. mTORi Could Enhance the Anti-Tumor Effects of CTGF/E7 DNA Vaccine to Extend the Survivals of Tumor-Bearing Mice

**Figure 7:**
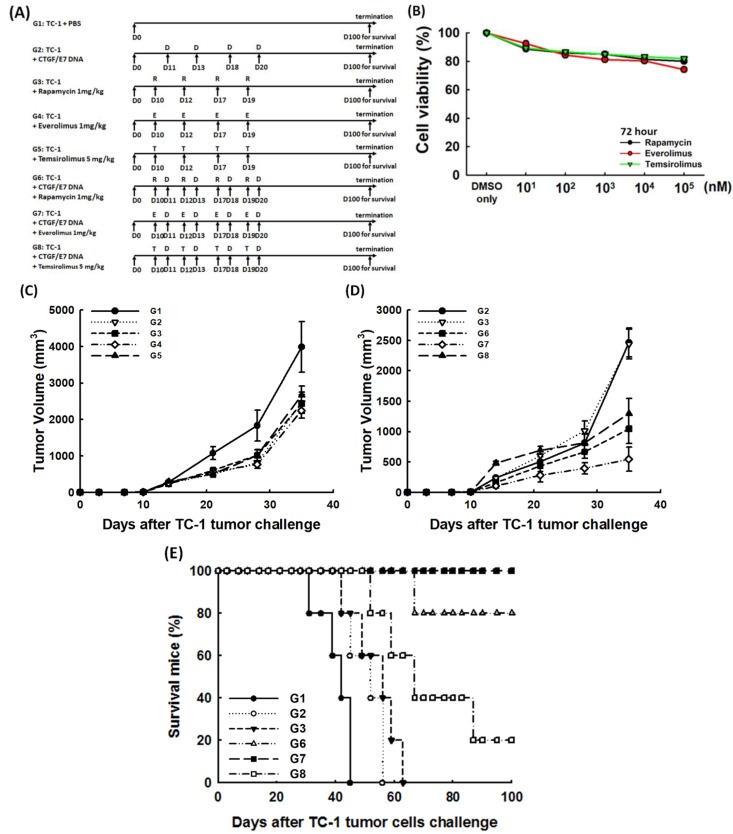

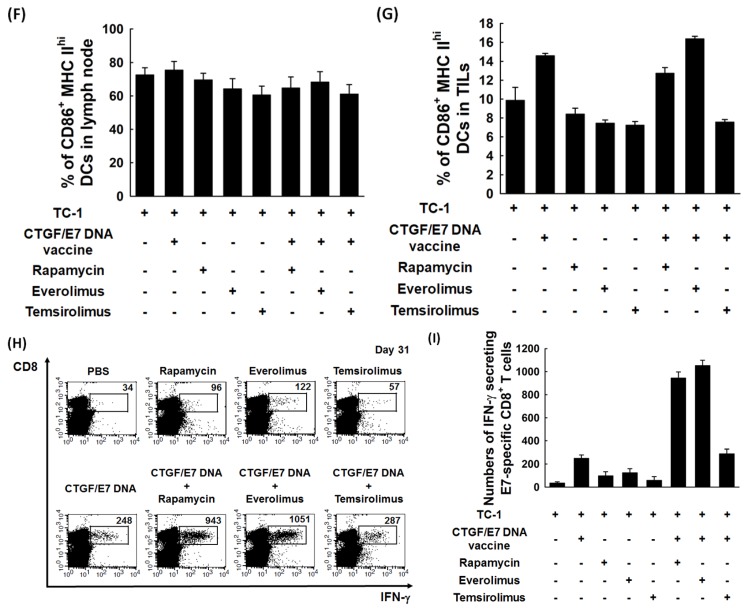
Anti-tumor effects and immune alteration of mice treated with CTGF/E7 DNA vaccine and/or mTORi. (**A**) Diagrammatic representation of different treatment protocols using CTGF/E7 DNA vaccine and/or mTORi. Note: G1: PBS only; G2: CTGF/E7 DNA vaccine; G3: Rapamycin; G4: Everolimus; G5: Temsirolimus; G6: CTGF/E7 DNA vaccine and rapamycin; G7: CTGF/E7 DNA vaccine and everolimus; G8: CTGF/E7 DNA vaccine and temsirolimus. (**B**) Effects of mTORi on TC-1 tumor cells. The cytotoxic effects of mTORi on TC-1 cells were not significant. (*n* = 3) (**C**,**D**) Tumor volumes in TC-1-bearing mice treated with different modalities using CTGF/E7 DNA vaccine and/or mTORi. On day 35 after tumor challenge, the mice treated with CTGF/E7 DNA vaccine and mTORi (G6–8) had less tumor volumes than other groups (G1–5) (*p* = 0.002, *n* = 2). (**E**) Survival analysis of mice in the various groups. All mice treated with CTGF/E7 DNA vaccine and everolimus (G7), 80% of mice treated with CTGF/E7 DNA vaccine and rapamycin (G6) and 20% of mice treated with CTGF/E7 DNA vaccine and temsirolimus (G8) were alive 100 days after TC-1 tumor challenge. However, none of the mice in the other groups (G1–3) survived more than 70 days of tumor challenge (*p* < 0.001, *n* = 2). (**F**) Bar figures exhibited the percentages of CD86^+^MHC II^hi^ DCs in tumor-associated draining lymph nodes of mice treated with the various protocols. When gated by the expression of CD11c and MHC I^hi^, the percentages of CD86^+^MHC II^hi^ DCs did not show differences among these groups (*p* = 0.14, *n* = 3). (**G**) Bar figures exhibited the percentages of CD86^+^MHC II^hi^ DCs in TILs of mice treated with the various protocols. When gated by the expression of CD11c and MHC I^hi^, the percentages of CD86^+^MHC II^hi^ DCs were highest in mice treated with CTGF/E7 DNA vaccine and everolimus (*p* = 0.027, *n* = 3). (**H**) Representative figures of flow cytometric analysis of E7-specific IFN-γ-secreting CD8^+^ cytotoxic T cells/3.5 × 10^5^ lymphocytes in tumor-associated draining lymph nodes in the various experimental groups. (*n* = 3) (**I**) Bar figures indicating numbers of E7-specific IFN-γ-secreting CD8^+^ T cells/3.5 × 10^5^ lymphocytes in tumor-associated draining lymph nodes in the various experimental groups. The number of E7-specific IFN-γ-secreting CD8^+^ T cells was highest in mice treated with CTGF/E7 DNA vaccine and everolimus (*p* = 0.003, *n* = 3).

To explore whether the combination of mTORi has a positive impact on generating more potent anti-tumor effects of CTGF/E7 DNA vaccine, various inhibitors of mTOR were used for in vivo tumor treatment via various therapeutic protocols (Figure 7A). The cytotoxic effects of mTORi on TC-1 cells were not significant (Figure 7B). On day 35 after tumor challenge, the mice treated with CTGF/E7 DNA vaccine and mTORi (G6: CTGF/E7 DNA vaccine and rapamycin, 1.0 ± 0.2 × 10^3^ mm^3^; G7: CTGF/E7 DNA vaccine and everolimus, 0.5 ± 0.2 × 10^3^ mm^3^; G8: CTGF/E7 DNA vaccine and temsirolimus, 1.3 ± 0.3 × 10^3^ mm^3^) had less tumor volumes than other groups (G1: PBS only, 4.0 ± 0.7 × 10^3^ mm^3^; G2: CTGF/E7 DNA vaccine, 2.5 ± 0.3 × 10^3^ mm^3^; G3: rapamycin, 2.4 ± 0.25 × 10^3^ mm^3^; G4: everolimus, 2.2 ± 0.2 × 10^3^ mm^3^; G5: temsirolimus, 2.7 ± 0.3 × 10^3^ mm^3^) (*p* = 0.002, Figure 7C,D). All mice treated with CTGF/E7 DNA vaccine and everolimus (G7), 80% of mice treated with CTGF/E7 DNA vaccine and rapamycin (G6) and 20% of mice treated with CTGF/E7 DNA vaccine and temsirolimus (G8) were alive 100 days after TC-1 tumor challenge. However, none of the mice in the other groups (G1–3) survived more than 70 days of tumor challenge (*p* < 0.001, Figure 7E). Tumor burden was the lowest in the surviving mice treated with CTGF/E7 DNA vaccine and everolimus (G7) 100 days after TC-1 tumor challenge (*p* = 0.002, Supplementary Figure S1).

Then, the percentages of mature DCs and antigen-specific immune responses in the TME were further in vivo analyzed. When gated by the expression of CD11c and MHC I^hi^, the percentages of CD86^+^MHC II^hi^ DCs in tumor-associated draining lymph nodes of mice treated with various protocols did not show differences (*p* = 0.14, Figure 7F). However, the percentages of CD86^+^MHC II^hi^ DCs in tumor-infiltrating lymphocytes (TILs) were highest in mice treated with CTGF/E7 DNA vaccine and everolimus (G7) (G1, 9.9 ± 1.4%; G2, 14.6 ± 0.3%; G3, 8.4 ± 0.6%; G4, 7.4 ± 0.3%; G5, 7.2 ± 0.4%; G6, 12.7 ± 0.6%; G7, 16.4 ± 0.3%; G3, 7.6 ± 0.3%; *p* = 0.027, Figure 7G). The number of E7-specific CD8^+^ T cells/3.5 × 10^5^ lymphocytes in tumor-associated draining lymph nodes analyzed by HPV-16 E7 tetramer staining was highest in mice treated with CTGF/E7 DNA vaccine and everolimus (G7) (*p* = 0.002, Supplementary Figure S2). 

Furthermore, Figure 7H showed the representative flow cytometric analysis of E7-specific IFN-γ-secreting CD8^+^ cytotoxic T cells/3.5 × 10^5^ lymphocytes in tumor-associated draining lymph nodes in the various experimental groups. The number of E7-specific IFN-γ-secreting CD8^+^ T cells/3.5 × 10^5^ lymphocytes was highest in mice treated with CTGF/E7 DNA vaccine and everolimus (G7) (G1, 34.3 ± 10.9; G2, 248.1 ± 29.9; G3, 96.2 ± 36.3; G4, 123.0 ± 35.8; G5, 57.5 ± 32.8; G6, 943.1 ± 54.3; G7, 1051.4 ± 47.1; G8, 287.8 ± 40.2; *p* = 0.003, Figure 7I).

## 3. Discussion

In this study, we evaluated in vitro the influences of mTORi on the DCs and in vivo investigated the potential application of mTORi to enhance the anti-tumor effects of DNA vaccine for cancer treatment. Suppressive effects of mTORi on the DC maturation markers expressed on BMMCs could be reversed. The mTORi-treated mature BMM-derived DCs tended to be non-apoptotic. These mTORi-treated BMM-derived DCs could have better antigen presenting and processing abilities. The tumoricidal activity of E7-specific cytotoxic CD8^+^ T lymphocytes could be increased following activation of mTORi-treated BMM-derived DCs. In addition, mTORi could strengthen the anti-tumor effects of CTGF/E7 DNA vaccine to extend the survivals of tumor-bearing mice.

DCs are pivotal antigen-presenting cells responsible for collecting, processing and presenting the tumor-associated or specific antigens on MHC class I and class II molecules and migrating to draining lymph nodes to initiate effector T lymphocytes [18]. DCs could induce not only anti-tumor T-cell responses in the lymphoid organs but also antibody and natural killer or natural killer T cell responses when the process of capture and presentation occurs under the stimulation of immunogenic maturation signals. The maturation signals could be provided endogenously by, for example, dying or necrotic tumor cells releasing factors, or exogenously by, for example, agonists of TLR [18,19]. However, DCs could induce tolerance leading to T-cell anergy or the production of regulatory T cells without these maturation stimuli [20,21,22,23].

In the previous studies, DC maturation can be induced by the TLR agonists but DC life span is significantly reduced following activation by these TLR ligands [6,7]. Our results also showed that LPS alone could induce DC maturation (Figure 2A–C), but the percentages of non-apoptotic cells in these LPS-activated BMM-derived DCs decreased with time (Figure 3B). Previous studies have shown that tumor cells and TME could prevent DC maturation and polarize DCs into immune suppressive status [24,25,26]. However, this study demonstrated TC-1 tumor cells could have the ability to induce DC maturation (Figure 2G) like other report [27], but the percentages of non-apoptotic cells in these mature BMM-derived DCs also still significantly decreased with time (Figure 3D). The reasons for tumor cells influencing DC maturation and early apoptosis might be associated with the soluble tumor-derived factors with immunomodulatory effects in tumor culture supernatant [27]. Therefore, extending the life span of mature DCs can potentially increase the number and duration of DCs in stimulating lymphocytes for enhancing immune responses.

Elongating the life span of LPS-activated mouse DCs to potentially increase the time available for T cell activation can be achieved by inhibiting mTOR to block the induction of NO production for a metabolic transition [7,28]. In our study, the results showed that the percentages of non-apoptotic cells in the LPS-stimulated BMM-derived DCs under the treatment of mTORi did not decrease with time and were higher in mTORi-treated groups than those in non-mTORi-treated group at indicated intervals (Figure 3B). In addition, this study demonstrated the percentages of non-apoptotic cells in TC-1 tumor cells-activated BMM-derived DCs decreased with time in non-mTORi-treated and mTORi-treated groups (Figure 3D). However, the percentages of non-apoptotic cells were still significantly higher in mTORi-treated groups than those in non-mTORi-treated groups at indicated intervals (Figure 3D).

Like previous report [7], the BMM-derived DCs pulsed with OVA peptides in the presence of mTORi were highly competent to in vitro present (Figure 4A,B) or process (Figure 4C,D) antigen. We further demonstrated that the antigen presenting (Figure 4E) and processing (Figure 4F) abilities of non-mTORi-treated BMM-derived DCs co-cultured with TC-1 tumor cells and then pulsed with OVA peptides would be lower than those only pulsed with OVA peptides. But, the activities of antigen presenting (Figure 4E) and processing (Figure 4F) of mTORi-treated BMM-derived DCs co-cultured with TC-1 tumor cells and then pulsed with OVA peptides were still better than those of non-mTORi-treated BMM-derived DCs only pulsed with OVA peptides. Higher tumor killing activity could be detected in TC-1/Luc tumor cells co-cultured with E7-specific CD8^+^ T lymphocytes activated by mTORi-treated BMM-derived DCs (Figure 5). In addition, the expression of apoptotic molecules, Bad or Bak can be inhibited in BMM-derived DCs treated with mTORi (Figure 6).

Cancer immunotherapy is designed to develop a highly active and durable population of tumor-specific T cells, but such therapeutic approaches have met with failure in most tumor types [29,30]. The primary reason can be that malignant cells can utilize a variety of pathways to escape immune surveillance [18,29]. To overcome these obstacles, strategically combining immunotherapies with other immuno-modulators to enhance anti-tumor effects is critical for treating cancers [31,32]. As shown in our study, the combination of mTORi has a positive impact on CTGF/E7 DNA vaccine to generate better tumor control (Figure 7C,D) and extend the survivals of tumor-bearing hosts (Figure 7E). Furthermore, the percentages of CD86^+^MHC II^hi^ mature DCs and antigen- specific immune responses in the TME were higher in the tumor-bearing hosts treated with combinational regimens (Figure 7F–I).

## 4. Materials and Methods

### 4.1. Mice

The female C57BL/6J mice (6–8 weeks old) were applied in this study. All mice were purchased and bred in the animal facility of the School of Medicine, National Taiwan University. All animal procedures were performed in accordance with the protocols approved by National Taiwan University College of Medicine and College of Public Health Institutional Animal Care and Use Committee (approval code: 20170392).

### 4.2. Cell Line

TC-1 and TC-1/Luc tumor cell production and maintenance have been previously described [33,34]. To generate TC-1 tumor cells, HPV16 E6, E7 and ras oncogene were used to transform primary C57BL/6 lung epithelial cells [33]. These TC-1 tumor cells were first transfected with GFP and luciferase and then isolated by flow cytometry to produce TC-1/Luc tumor cells as previously described [33].

### 4.3. Preparation of DNA Construct and DNA Bullet

pcDNA3-CTGF/E7 plasmid was prepared as previously described [35]. For DNA bullet preparation, nanogold particles were first coated with pcDNA3-CTGF/E7 plasmids. The DNA vaccine was delivered via a low pressure-accelerated gene gun (BioWare Technologies, Taipei, Taiwan). The protocol of DNA vaccinations was subcutaneously delivered by gene gun twice per week for two weeks since day 11 at the bilateral axillary and inguinal areas, based on the locations of draining lymph nodes and avoiding the subcutaneous TC-1 tumor [36].

### 4.4. Generation of Immature BMM-Derived DCs

The BMMCs were harvested as previously described [37]. BMMCs acquired from the femurs of C57BL/6J mice were cultured for 6 days and refreshed medium for every two days. The cells were seeding at a density of 1 × 10^6^ cells/mL in 24-well plates (Sarstedt, Newton, NC, USA) in a total volume of 2 mL/well with 10 ng/mL of recombinant murine GM-CSF (PeproTech, Rocky Hill, NJ, USA) to generate immature BMM-derived DCs.

For the experiments of BMMCs treated with/without mTORi, BMMCs were harvested and cultured as described above and the mTORi (rapamycin: 10 nM; everolimus: 10 nM; temsirolimus: 2 µM) were also treated from day 1 to day 6. These cells were used for the following experiments.

For the experiments of immature BMM-derived DCs treated with/without mTORi, BMMCs were cultured to generate immature BMM-derived DCs as described above and then treated with mTORi (rapamycin: 10 nM; everolimus: 10 nM; temsirolimus: 2 µM) for 24 h. These cells were used for the following experiments.

### 4.5. Maturation of BMM-Derived DCs Stimulated by LPS, CTGF/E7 DNA Plasmid and TC-1 Tumor Cells

For the stimulated maturation of immature BMM-derived DCs, there were three different ways in this study. BMMCs were harvested and cultured to develop immature BMM-derived DCs as described above. The LPS (4 ng/mL, 20 ng/mL and 100 ng/mL) and CTGF/E7 DNA plasmid (0.2 µg/mL, 1 µg/mL and 5 µg/mL) with various doses were added in immature BMM-derived DCs on day 6 for 24 h [38]. For the stimulated maturation of immature BMM-derived DCs by tumor cells, immature BMM-derived DCs were co-culture with TC-1 tumor cells at the ratio of BMMC:TC-1 = 1:1 on day 6 for 24 h to induce maturation [39].

### 4.6. Flow Cytometric Analysis of the Surface Markers of BMM-Derived DCs

The BMM-derived DCs were generated as describe above and stained with FITC-conjugated anti-CD11c (eBioscience, Thermo Fisher Scientific, San Diego, CA, USA), PE-conjugated anti-CD11c (eBioscience), PE-conjugated anti-MHC class I (eBioscience), PE-Cy5.5-conjugated anti-CD86 (BioLegend, San Diego, CA, USA) or APC- conjugated anti-MHC class II (BioLegend) Ab. To evaluate the percentages of mature DCs in the draining lymph nodes and tumors, the cells expressed with CD11c^+^ and MHC class I^hi^ were gated as dendritic cells and then analyzed the co-expression of CD86^+^ and MHC class II^hi^. Flow cytometric analyses were performed as previously described [37,40].

### 4.7. In Vitro Apoptosis Assay of BMM-Derived DCs by Flow Cytometric Analyses

To detect the viability of the BMM-derived DCs, these cells were stained with 7-AAD and annexin V and analyzed by flow cytometry. In brief, the mature BMM-derived DCs were prepared as described above and then harvested for apoptotic assay. Immature BMM-derived DCs treated with DMSO were used as control group. These cells were then incubated with 7-AAD and FITC-conjugated annexin V (BD Bioscience, Franklin Lakes, NJ, USA) and analyzed by flow cytometry [41].

### 4.8. In Vitro Antigen Presentation Ability of BMM-Derived DCs Treated with mTORi

To analyze the influence of mTORi on antigen presentation ability, the BMMCs were treated with different mTORi (rapamycin: 10 nM; everolimus: 10 nM; temsirolimus: 2 µM) or DMSO (control group) to generate immature BMM-derived DCs. 1 μg/mL FITC-conjugated OVA short peptides (OVA_257-264_ [SIINFEKL]) (Invitrogen, Thermo Fisher Scientific, San Diego, CA, USA) were co-cultured with these cells on day 6 for 3 h as previously described [42].

To investigate whether the mTORi could restore the antigen presentation ability of BMM-derived DCs inhibited by tumor cells, BMM-derived DCs were co-cultured with TC-1 tumor cells and then pulsed with FITC-conjugated OVA short peptide. In brief, the BMMCs were treated with different mTORi (rapamycin: 10 nM; everolimus: 10 nM; temsirolimus: 2 µM) or DMSO (control group) to generate immature BMM-derived DCs. These cells were co-cultured with TC-1 tumor cells for 6 h and then pulsed with 1 μg/mL FITC-conjugated OVA short peptides for 3 h. All cells were stained with PE-conjugated anti-CD11c Ab (eBioscience) and evaluated by flow cytometry.

### 4.9. In Vitro Antigen Processing Ability of BMM-Derived DCs Treated with mTORi

To analyze the influence of mTORi on antigen processing abilities, the BMMCs were treated with different mTORi (rapamycin: 10 nM; everolimus: 10 nM; temsirolimus: 2 µM) or DMSO (control group) to generate immature BMM-derived DCs. 10 μg/mL FITC-conjugated OVA long peptides (OVA_323–339_ [ISQAVHAAHAEI- NEAGR]) (Invitrogen) were co-cultured with these cells on day 6 for 3 h as previously described [42].

To investigate whether the mTORi could restore the antigen processing ability of BMM-derived DCs inhibited by tumor cells, BMM-derived DCs were co-cultured with TC-1 tumor cells and then pulsed with FITC-conjugated OVA long peptide. Briefly, the BMMCs were treated with different mTORi (rapamycin: 10 nM; everolimus: 10 nM; temsirolimus: 2 µM) or DMSO (control group) to generate immature BMM-derived DCs. These cells were co-cultured with TC-1 tumor cells for 6 h and then pulsed with 10 μg/mL FITC-conjugated OVA long peptides for 3 h. All cells were stained with PE-conjugated anti-CD11c Ab (eBioscience) and analyzed by flow cytometry.

### 4.10. In Vitro Anti-Tumor Activity of E7-Specific Cytotoxic CD8^+^ T Lymphocytes Activated by BMM-Derived DCs Treated with mTORi

In vitro anti-tumor killing assay was performed as previously described [42]. The BMMCs were treated with different mTORi (rapamycin: 10 nM; everolimus: 10 nM; temsirolimus: 2 µM) or DMSO (control group) to generate immature BMM-derived DCs. These DCs (1 × 10^5^ cells/well) were pulsed with 1 μg/mL Db-compatible MHC I E7 peptide (aa49–57) on day 6 and then co-cultured with the E7-specific CD8^+^ T cell line (1:10 ratio) overnight. The activated E7-specific CD8^+^ T cells were then co-cultured with the TC-1/Luc (1:10 ratio) in a 96-well plate (1 × 10^4^ cells/well) for 24 h. Luciferin (Promega, Madison, WI, USA) was added and the luciferase activities of tumor growth were measured using IVIS^®^ Imaging Systems (Xenogen/Caliper Life Sciences Inc., Hopkinton, MA, USA).

### 4.11. Western Blot Analysis of BMM-Derived DCs Treated with mTORi

To analyze the signaling transduction pathways, western immunoblotting was performed as previously described [42]. Briefly, the BMMCs were treated with different mTORi (rapamycin: 10 nM; everolimus: 10 nM; temsirolimus: 2 µM) or DMSO (control group) to generate immature BMM-derived DCs and then co-cultured with TC-1 tumor cells on day 6 for 6 h. All groups were lysed in PhosphoSafe™ Extraction Reagent (Novagen, Merck KGaA, Darmstadt, Germany) with a proteinase inhibitor (Sigma, Merck KGaA, Darmstadt, Germany). The protein extracts were quantified with a BCA Protein Assay Kit (Pierce, Rockford, IL, USA).

These targeting proteins were separated by SDS/PAGE (10% gel), transferred onto an PVDF membrane (Millipore, Merck KGaA), and probed with primary antibodies specific to mTOR (Genetex, Irvine, CA, USA), phospho-mTOR (Genetex), Bad (Genetex), Bak (Genetex), p38 (BioLegend), phospho-p38 (Millipore) and GAPDH (Abcam, Cambridge, MA, USA). Then, HRP-conjugated second antibodies (Hycult Biotech, Uden, The Netherlands) were probed and the specific bands were visualized by ECL^®^ Western blotting system (GE Healthcare, Salt lake city, UT, USA). The band density was quantified with ImageJ and the relative ratios of the indicated proteins/GAPDH were shown.

### 4.12. In Vivo Tumor Treatment

The different treatment regimens for CTGF/E7 DNA vaccines and/or mTORi were shown in Figure 7A. The mTORi would be diluted to assumed concentration (rapamycin: 1 mg/kg; everolimus: 1 mg/kg; temsirolimus: 5 mg/kg) and intraperitoneally administered to mice. Briefly, C57BL/6J mice (10 mice per group) were subcutaneously challenged with 5 × 10^4^ TC-1 tumor cells on day 0. Tumors were measured with digital callipers and approximated with the largest diameter (α) of the tumor, using the formula: volume=α^3^/2. Tumors were allowed to grow for 10 days with approximately 5–7.5 mm^3^ in volume. These tumor-bearing mice were randomized into groups such that each treatment group would have similar average tumor sizes. On day 10, the tumor-bearing mice were treated with different mTORi twice per week for 2 weeks. On day 11, the tumor-bearing mice were treated with 2 µg CTGF/E7 DNA vaccine twice per week for two weeks. Mice were sacrificed on day 31 after tumor challenge for immunologic profiling assays (5 in each group). The remaining animals (5 in each group) were sacrificed when the tumor volume reached >4000 mm^3^, or kept until 100 days after tumor challenge or death for the survival analysis. The tumor sizes in the different groups were recorded and compared.

### 4.13. MTT Cytotoxicity Assay

To test the cytotoxic effects of mTORi on TC-1 tumor cell, the MTT assay was performed [43]. In brief, TC-1 tumor cells were seeded in 96 well plates at 2 × 10^3^ per well and treated with various doses (10^1^ nM, 10^2^ nM, 10^3^ nM, 10^4^ nM and 10^5^ nM) of different mTORi for 72 h. Four h before the end of experiments, 30 μL of 3-(4,5-dimethylthiazol-2-yl)-2,5-diphenyl-2*H*-tetrazolium bromide (MTT) (0.5 mg/mL) were added to each well. The culture medium was removed and the cells were solubilized in DMSO 200 µL/well at the end of the experiment. The absorbance of each well was determined using a microplate reader with a test wavelength of 570 nm and a reference wavelength of 630 nm.

### 4.14. Preparation of Lymphocytes from Tumor-Associated Draining Lymph Nodes and Tumors

TC-1 tumor-bearing mice were treated with CTGF/E7 DNA vaccine and/or mTORi as described above and sacrificed on day 31 after tumor challenge for immunologic assays. The protocol for isolating lymphocytes from the tumor-associated draining lymph nodes was the same as that for preparing splenocytes from the spleens. Therefore, the lymphocytes in draining lymph nodes and tumors can be obtained from mice with various treatment modalities as previously described [36,40].

### 4.15. Intracellular Interferon-γ Cytokine and MHC I-Restricted E7 peptide H-2D^b^ Tetramer Staining by Flow Cytometric Analysis

The lymphocytes from draining lymph nodes and tumors of various experimental groups were incubated with 1 μg/mL MHC I-restricted E7 peptide (aa49–57) as previously described [36,40]. We performed cell surface marker staining with PerCP/Cy5.5-conjugated anti-CD8 (Biolegend) and then intracellular staining with FITC-conjugated anti-mouse interferon-γ (Biolegend). The lymphocytes were also stained with FITC-conjugated anti-CD8a Ab (Abcam) and APC-conjugated H-2D^b^/E7_49–57_ tetramers (MBL International Corporation, Woburn, MA, USA). These cells were analyzed by flow cytometry [36,40].

### 4.16. Statistical Analysis

SPSS for Windows version 15.0 (SPSS Inc., Chicago, IL, USA) was applied for all statistical analyses. The in vivo and in vitro data were shown as mean ± SE (standard error), which represented at least two different experiments. The results of flow cytometry, luminescence, and tumor sizes were evaluated with the Kruskal-Wallis test. In the survival experiments, the event time distributions were analyzed by Kaplan-Meier method and log rank test. Numbers (*n*) of the experimental repeats for statistical analysis were presented. Survival analysis and tumor measurement of tumor-bearing mice in the various experimental groups were performed in duplicate. The remaining experiments were performed in triplicate. A *p* < 0.05 was defined as statistically significant.

## 5. Conclusions

In conclusion, our study in vitro demonstrated that mTOR inhibition can extend the life span and enhance the antigen presenting and processing abilities of BMM-derived DCs even in the presence of tumor cells. The tumor-bearing hosts treated with DNA vaccine combined with mTORi indeed can have higher percentages of mature DCs in the TME, better disease control and longer survivals. Therefore, application of mTORi can be a pharmacological approach for temporally increasing life span, antigen presenting and antigen processing of DCs to strengthen the therapeutic outcome of cancer immunotherapy.

## Supplementary Materials

The following are available online at https://www.mdpi.com/2072-6694/11/5/617/s1, Figure S1: Tumor volumes of surviving mice 100 days after tumor challenge in the various experimental groups, Figure S2: Numbers of E7-specific CD8^+^ T cells in tumor-associated draining lymph nodes in the various experimental groups.
