# Supplementary material for: mTOR Inhibitors Can Enhance the Anti-Tumor Effects of DNA Vaccines through Modulating Dendritic Cell Function in the Tumor Microenvironment
Source: Cancers (Basel). 2019 May 2;11(5):617. doi: 10.3390/cancers11050617 (PMC6562783; doi:10.3390/cancers11050617)
Supplement: Supplementary file 1 [file cancers-11-00617-s001.zip › cancers-496714-supplement-final/cancers-496714-supplementary figures 1 and 2.pdf]

Supplementary Materials

mTOR Inhibitors Can Enhance the Anti-Tumor Effects of DNA Vaccines through Modulating Dendritic Cell Function in the Tumor Microenvironment

Yu-Li Chen, Han-Wei Lin, Nai-Yun Sun, Jr-Chi Yie, Hsueh-Chih Hung, Chi-An Chen, Wei-Zen Sun and Wen-Fang Cheng

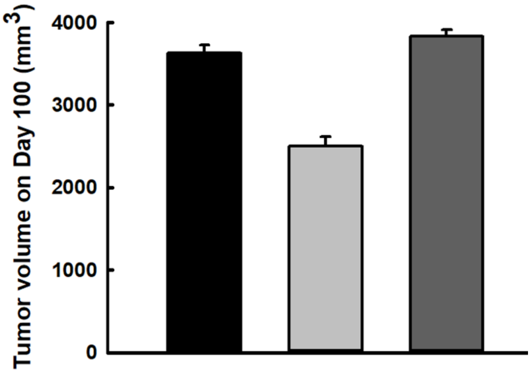

Figure S1. Tumor volumes of surviving mice 100 days after tumor challenge in the various experimental groups. Tumor burden was the lowest in the surviving mice treated with CTGF/E7 DNA vaccine and everolimus (G7) (G6, 3627.6 ± 95.9 mm^3; G7, 2497.6 ± 116.7 mm^3; G8, 3826.8 ± 86.1 mm^3; p = 0.002, n = 2).

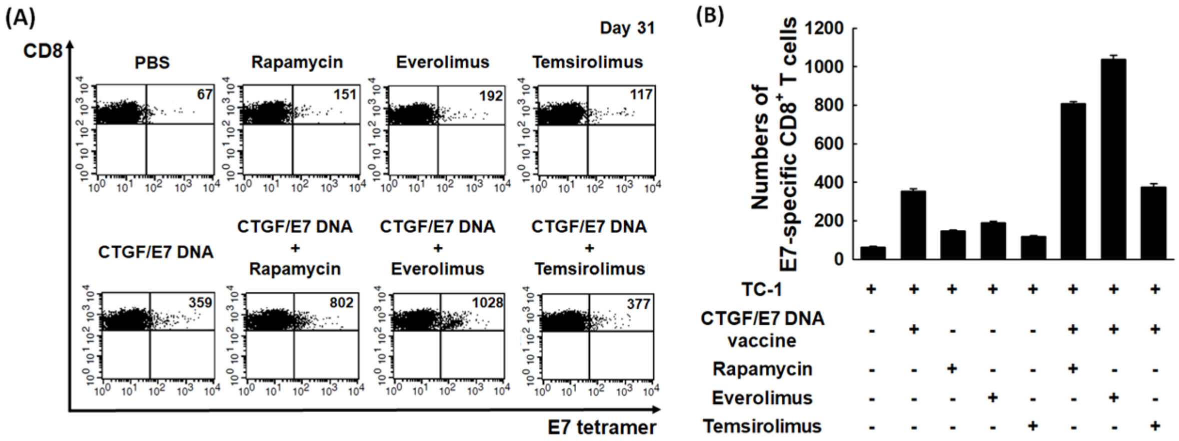

Figure S2. Numbers of E7-specific CD8⁺ T cells in tumor-associated draining lymph nodes in the various experimental groups. (**A**) Representative flow cytometric analysis of E7-specific CD8⁺ cytotoxic T cells/3.5 × 10^5 lymphocytes in tumor-associated draining lymph nodes in the various experimental groups by HPV-16 E7 tetramer staining. (n = 3) (**B**) Bar figures indicating numbers of E7-specific CD8⁺ T cells/3.5 × 10^5 lymphocytes in tumor-associated draining lymph nodes in the various experimental groups by HPV-16 E7 tetramer staining. The number of E7-specific CD8⁺ T cells/3.5 × 10^5 lymphocytes was highest in mice treated with CTGF/E7 DNA vaccine and everolimus (G7) (G1, 62.0 ± 4.4; G2, 352.3 ± 13.1; G3, 145.7 ± 5.5; G4, 189.0 ± 8.9; G5, 116.7 ± 6.5; G6, 806.3 ± 11.6; G7, 1038.7 ± 21.1; G8, 374.0 ± 18.9; p = 0.002, n = 3).
