# Supplementary material for: mTOR Inhibitors Can Enhance the Anti-Tumor Effects of DNA Vaccines through Modulating Dendritic Cell Function in the Tumor Microenvironment
Source: Cancers (Basel). 2019 May 2;11(5):617. doi: 10.3390/cancers11050617 (PMC6562783; doi:10.3390/cancers11050617)
Supplement: Supplementary file 1 [file cancers-11-00617-s001.zip › cancers-496714-supplement-final/Original Data of western blot (Figure 6).pptx]

## Slide 1
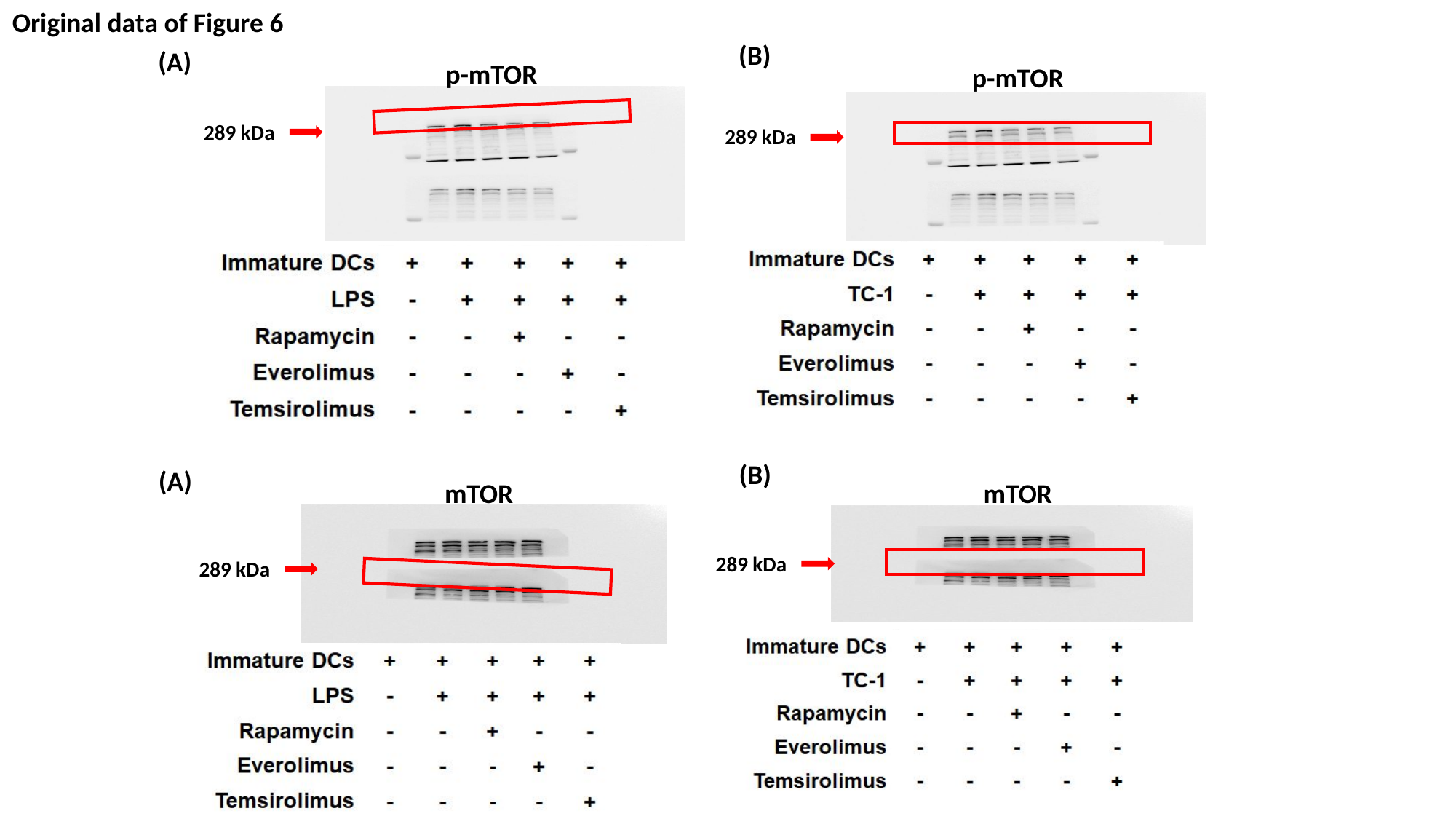

Original data of Figure 6
(B)
(A)
p-mTOR
p-mTOR
289 kDa
289 kDa
(B)
(A)
mTOR
mTOR
289 kDa
289 kDa

## Slide 2
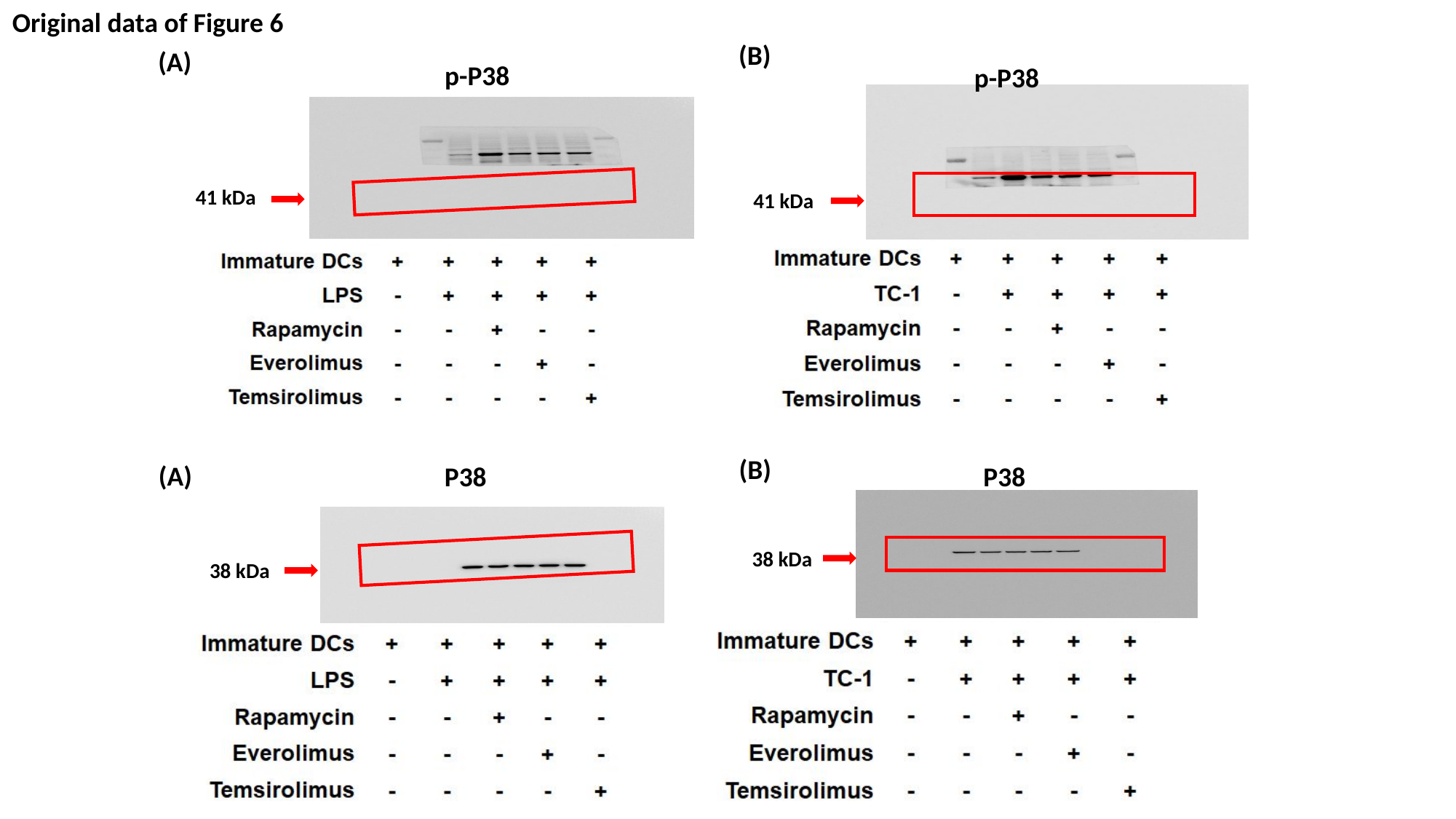

Original data of Figure 6
(B)
(A)
p-P38
p-P38
41 kDa
41 kDa
(B)
(A)
P38
P38
38 kDa
38 kDa

## Slide 3
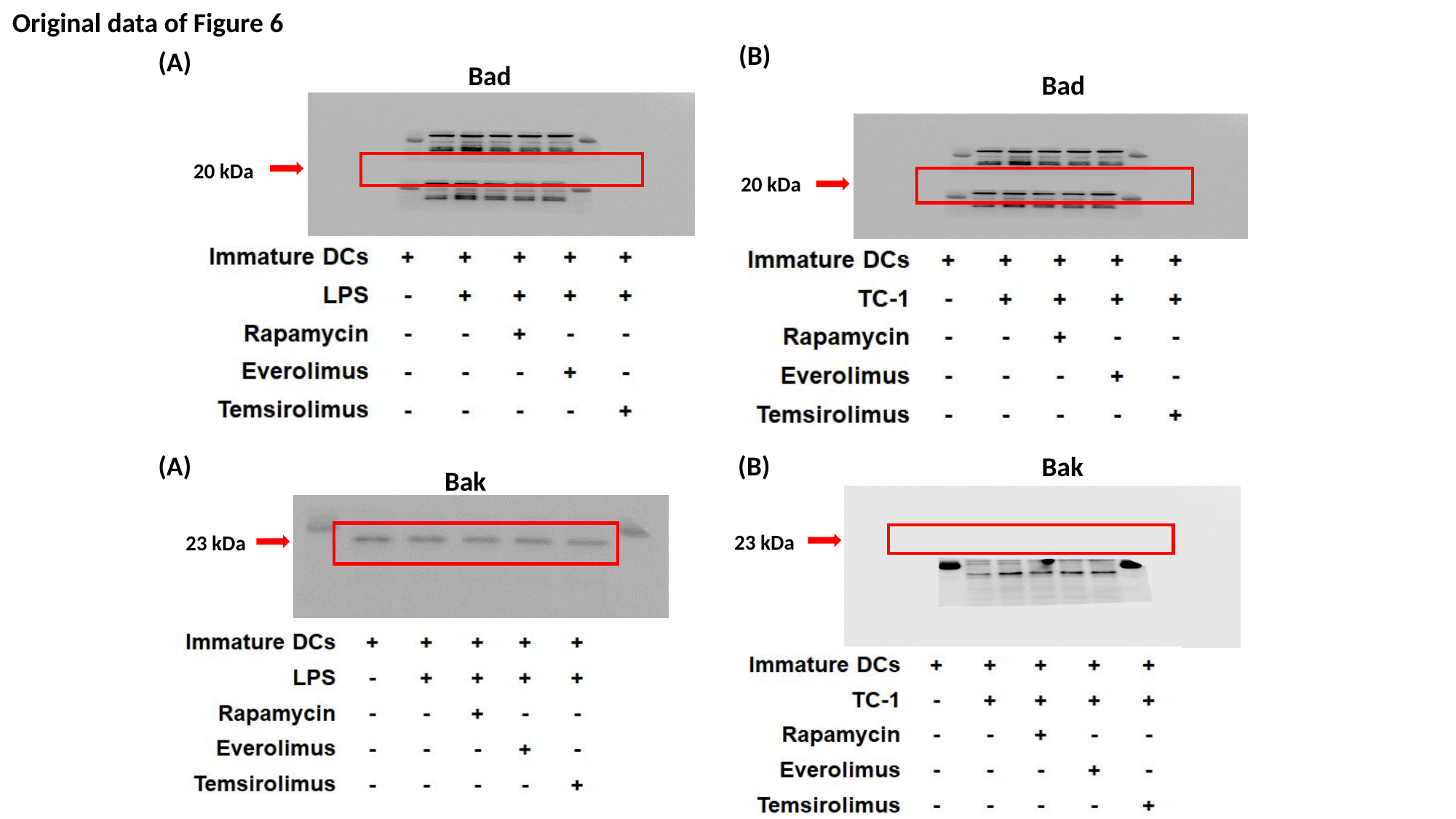

Original data of Figure 6
(B)
(A)
Bad
Bad
20 kDa
20 kDa
(A)
(B)
Bak
Bak
23 kDa
23 kDa

## Slide 4
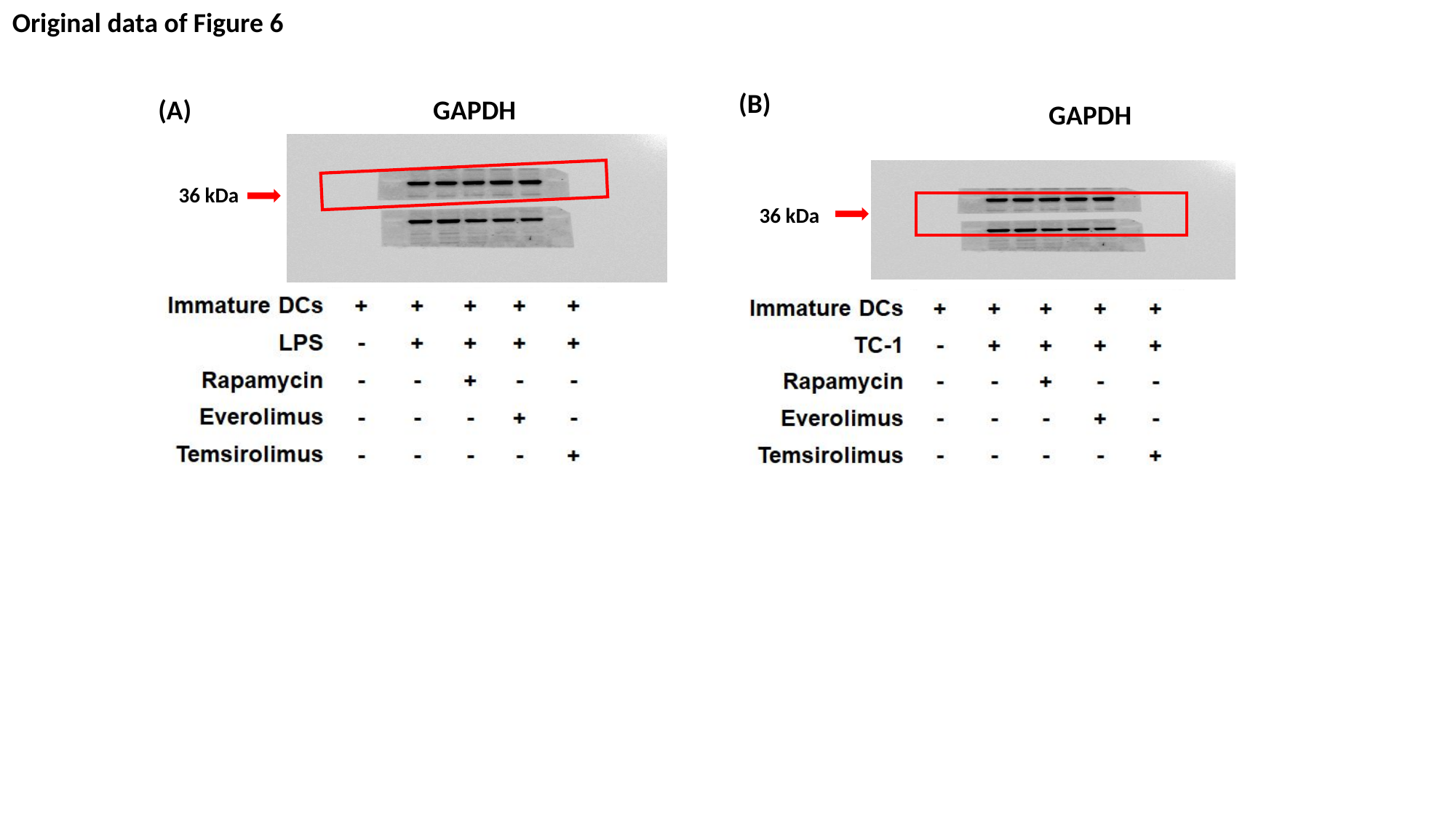

Original data of Figure 6
(B)
(A)
GAPDH
GAPDH
36 kDa
36 kDa
.
